# Pro-Anorexia and Pro-Recovery Photo Sharing: A Tale of Two Warring Tribes

**DOI:** 10.2196/jmir.2239

**Published:** 2012-11-07

**Authors:** Elad Yom-Tov, Luis Fernandez-Luque, Ingmar Weber, Steven P Crain

**Affiliations:** ^1^Microsoft ResearchHerzliyaIsrael; ^2^Northern Research InstituteTromsøNorway; ^3^Computer Science DepartmentUniversity of TromsøTromsøNorway; ^4^Yahoo ResearchBarcelonaSpain; ^5^Department of Computer ScienceOberlin CollegeOberlin, OHUnited States

**Keywords:** Medical Informatics, Internet, Photo, Eating Disorder, Anorexia Nervosa, Social Network

## Abstract

**Background:**

There is widespread use of the Internet to promote anorexia as a lifestyle choice. Pro-anorexia content can be harmful for people affected or at risk of having anorexia. That movement is actively engaged in sharing photos on social networks such as Flickr.

**Objective:**

To study the characteristics of the online communities engaged in disseminating content that encourages eating disorders (known as “pro-anorexia”) and to investigate if the posting of such content is discouraged by the posting of recovery-oriented content.

**Methods:**

The extraction of pro-anorexia and pro-recovery photographs from the photo sharing site Flickr pertaining to 242,710 photos from 491 users and analyzing four separate social networks therein.

**Results:**

Pro-anorexia and pro-recovery communities interact to a much higher degree among themselves than what is expected from the distribution of contacts (only 59-72% of contacts but 74-83% of comments are made to members inside the community). Pro-recovery users employ similar words to those used by pro-anorexia users to describe their photographs, possibly in order to ensure that their content appears when pro-anorexia users search for images. Pro-anorexia users who are exposed to comments from the opposite camp are less likely to cease posting pro-anorexia photographs than those who do not receive such comments (46% versus 61%), and if they cease, they do so approximately three months later. Our observations show two highly active communities, where most interaction is within each community. However, the pro-recovery community takes steps to ensure that their content is visible to the pro-anorexia community, both by using textual descriptions of their photographs that are similar to those used by the pro-anorexia group and by commenting to pro-anorexia content. The latter activity is, however, counterproductive, as it entrenches pro-anorexia users in their stance.

**Conclusions:**

Our results highlight the nature of pro-anorexia and pro-recovery photo sharing and accentuate the need for clinicians to be aware of such content and its effect on their patients. Our findings suggest that some currently used interventions are not useful in helping pro-anorexia users recover. Thus, future work should focus on new intervention methods, possibly tailored to individual characteristics.

## Introduction

### Background

Eating disorders, such as anorexia nervosa and bulimia, are highly prevalent in most developed countries [1]. These disorders are a major public health concern due to their high mortality and co-morbidities [2]. Anorexia nervosa commonly appears during adolescence and can be devastating to patients’ well-being and eventually may cause death [1,2].

Although generally viewed as highly negative by the general public, there is an online movement of people promoting anorexia as a lifestyle choice [3,4]. These communities are variously referred to as pro-anorexia, pro-ana, or simply “ana”. Several studies of content published on pro-anorexia websites and forums have identified the following characteristics of pro-anorexia communities [3-5]. Members of these communities use particular websites to share photographs and text designed to inspire members to lose weight (or maintain an immoderately low weight), as well as tips on how to lose weight to extremes that are far beyond reasonable. These websites also frequently operate forums where members can discuss issues related to their positive view of anorexia and to support each other in maintaining their disease (known as having a “pro-ana buddy”) [5]. As explained below, pro-anorexia content can be harmful and it is a public health concern.

An increasing percentage of the population in Europe and America use the Internet to find health-related information [6,7]. Unfortunately, not all the online health-related information is trustworthy or harmless. In addition to pro-anorexia online content, there are examples of online content overemphasizing possible harmful effects of vaccinations [8], reinforcing self-injury behavior [9], and even teaching asphyxiation techniques [10]. The rise of harmful content is a serious concern, since it is possible that people searching for trustworthy information (for example, healthy weight loss) may come across pernicious content (such as tips about inducing vomits). Furthermore, the use of online content promoting anorexia as a lifestyle is highly prevalent [11,12]. Custers et al found that 12% of Belgian female students in 6th, 9th and 11th grade had viewed pro-anorexia content [12]. Consumption of pro-anorexia content has been found to correlate with worsening of anorexia [11,13,14]. Rouleau et al in a recent review described three potential risks associated with pro-anorexia webs: the prevention of help-seeking, reinforcement of disordered eating, and operating under the guise of support [14].

Online pro-anorexia content is a case of societal concern and some health authorities and organizations have been attempting to curb its effects. For example, the Israeli government promoted the banning of advertisements that show severely underweight models [15]. Driven by public pressure, social content-sharing sites such as tumblr.com and pintrest.com have attempted to ban pro-anorexia content [16]. Lewis et al suggested intervening in the search results in order to prevent people from accessing pro-anorexia content [17]. Finally, warning labels have been added by the Internet Service Provider (ISP) when users try to access pro-anorexia content [18]. Except for the latter (which has been shown effective in reducing the risk to exposure to such content), it is unclear if these interventions are effective. One should also note that all these interventions are likely to be more effective at preventing new users from starting to engage with pro-anorexia content than in dissuading existing users from continuing to consume such content.

The pro-anorexia online communities have come under scrutiny by several research groups. Norris et al conducted an in-depth analysis of 12 pro-anorexia websites and found that the most prevalent themes in them were related to inspiration and assistance in achieving or maintaining anorexia [3]. A more recent study examined 180 pro-anorexia websites and found similar results [4]. Mulveen examined 15 discussion threads on a pro-anorexia site to discover the main themes of the discussion and to understand the reasons for participation in such threads [5]. However, the small number of threads investigated makes it difficult to draw conclusions from this investigation. In addition, there is a lack of studies addressing the characteristics of the pro-anorexia communities in comparison with those that featured anorexia simply as a disease.

Previous studies were relatively limited in the volume of data they examined, as well as in the fact that they focused solely on the pro-anorexia communities. However, the study of the different anorexia-related communities is highly relevant since these communities are dynamic and members are expected to change from pro-anorexia to pro-recovery communities and vice versa. In our study, we examined a very large body of pro-anorexia and pro-recovery data.

Our data were extracted from a social sharing site, which allows users to create multiple social networks. Thus, beyond an examination of the pro-anorexia and pro-recovery communities, we investigated the interactions between them. Following Wilson et al [11], we define pro-anorexia sites as those encouraging disordered eating. Pro-recovery sites are those that express a recovery-oriented perspective. Both sites, of which the former are more numerous [11], include individual expressions and community tools (such as discussion boards). In our study, we defined a pro-anorexia user as one who is actively involved in the creation and dissemination of content that takes a positive and encouraging attitude towards eating disorders, anorexia nervosa in particular. Conversely, pro-recovery users are those who share views of eating disorders as diseases from which one will want to recover and that take a negative view of them.

In addition, there is a lack of knowledge of the different pro-anorexia communities that are emerging in general content-based social networks. For example, to the best of our knowledge there are no studies on pro-anorexia videos disseminated on sites such as YouTube. In addition, image-sharing platforms, such as Flickr and Pinterest, are very popular and form a perfect venue for the dissemination of pro-anorexia photos (eg, ultra thin models) [16]. In this study, we focus on the study of anorexia-related communities on Flickr, which is an image-sharing platform supported by Yahoo Inc. Flickr is one of the most popular photo-sharing websites with nearly 80 million monthly visitors worldwide [19,20]. Moreover, as described below, several kinds of social connections are induced within the site, allowing for a thorough analysis of social effects within the site.

### Objectives

We aimed to study anorexia-related communities on Flickr, which is an image-sharing platform. Our main hypothesis is that there are two different and interrelated pro-anorexia and pro-recovery communities within Flickr. Specifically, our aims are: (1) To understand the community dynamics of the pro-anorexia and pro-recovery communities, and (2) To investigate why pro-recovery users post their content and to understand if this facilitates the recovery of pro-anorexia users.

## Methods

### Materials

**Figure 1:**
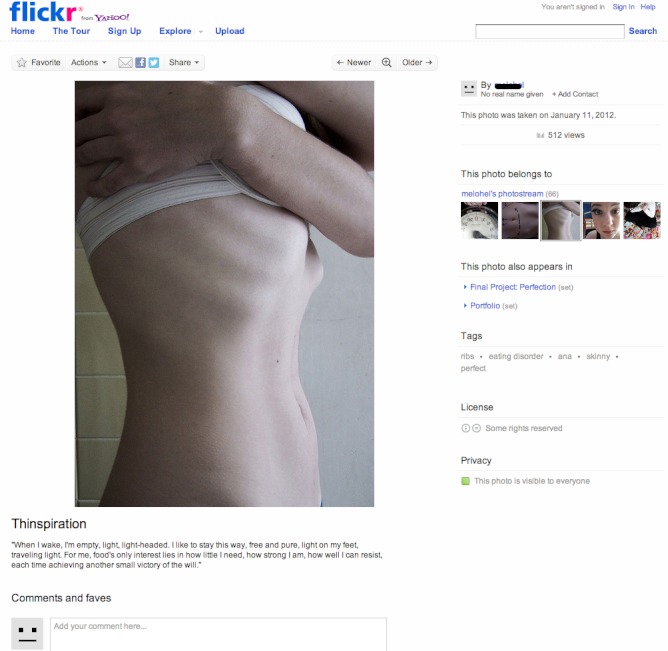
Example of a pro-anorexia image on Flickr [21].

**Figure 2:**
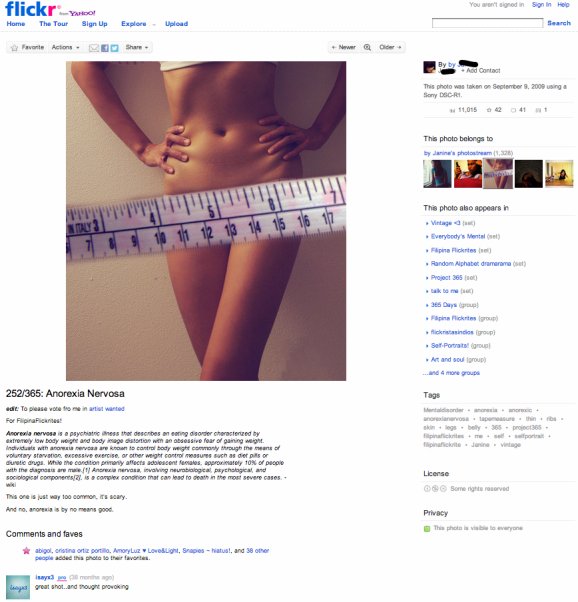
Example of a pro-recovery image on Flickr [22].

We used data from Yahoo’s photo-sharing site Flickr. Our data comprises four kinds of links: Contacts, favorites, comments, and tags. See Figure 1 for an example of an image and its annotations from the pro-anorexia community [21]. The photograph is shown on the top left hand side. A textual description is below it. Comments by other users are shown below it, as are markings showing if this photograph is chosen as a “favorite” by any user. On the right hand side are the tags used to describe the image. Figure 2 is an example from the pro-recovery community [22].

Flickr users can post both public and private data. In our analysis, we used data that was public on Flickr during February 2012. Most data were obtained through the Flickr API [23] except for information on who “favorited” a particular image, which was obtained by crawling actual pages.

We identified a set of (potentially) pro-anorexia and pro-recovery users using four methods:

1. Searching for photos that matched at least one of the search terms: “thinspo”, “thinspiration”, or “pro-ana”. Matches could occur in the tags, the photo title, or its description. All users who uploaded at least two such photos and whose profile still existed were added to the set of seed users. There were 162 such users.
2. Finding all users who uploaded at least two photographs to the anorexia-related Flickr groups “Eating Disorders Art” [24], “Anorexia Nervosa” [25], “Anorexia Help” [26], and “ED Healing” [27]. There were 71 such users.
3. Finding all users who commented on at least two photos that had one of the aforementioned tags or that was posted in one of the aforementioned groups. There were 669 such users.
4. Finding all users whose profile still existed who favorited (ie, marked as “favorite”) at least two photos that were tagged as both “thinspo” and “skinny” as well as one of “pretty”, “cute”, or “beautiful”. There were 14 such users.

For each of these users, we obtained the information on their activity on the Flickr site as follows:

1. Photo meta-information: We obtained meta-information for the 5000 most recent photos posted by each user. This information includes title, tags, description, date posted, the number of times that the photo was viewed, as well as geographic location information, where available. In total, we obtained information for 543,891 photos.
2. Comments for photos: We extracted comments for the 500 most recent photos of each user. This information includes the comment text, an identifier of who left the comment and the time stamp of when it was left. In total, we gathered 2,229,489 comments left for 106,877 photos uploaded by 739 users.
3. Favoriting of photos: We extracted the list of users who marked each of the 500 most recent photos of each user. In total, there were 642,317 such instances, pertaining to 88,337 photos uploaded by 753 users.
4. Public contacts: The list of contacts of each user was obtained. In total, the (directed) contact graph contained 237,165 outgoing edges for 721 seed users. Of these, 2821 edges were between two seed users, pointing to 543 distinct users.

Five researchers independently labeled the users to their degree of pro-anorexia or pro-recovery stance. The researchers were asked to classify users according to their support of pro-anorexia or pro-recovery content using a Likert-based scale. The labels provided by the researchers were averaged and used to classify the users into different classes. Kappa agreement in labeling was 0.51 (*P*< .001) [28]. Thus, good agreement is achieved on the view of users. Following labeling, our data consisted of 172 pro-recovery users and 319 pro-anorexia users. Anecdotally, while many pro-anorexia users self-identify as having an eating disorder, pro-recovery users rarely self-identify, and when they do, we found that approximately 19% of users identify as having formerly suffered from an eating disorder.

We identified tags related to anorexia content (both pro-anorexia and pro-recovery) by representing the tags using a vector space model and selecting all tags that appeared solely in this content or were at least 10 times more likely compared to content that was identified as neutral by the labelers. A total of 25,689 photographs, which we refer to as “highly relevant” photos, contained at least one of these tags.

Processing of data was performed using Matlab 7.3, graph layouts were performed with Gephi 0.8.1. All research described herein was approved by the Yahoo internal research board on human research and consisted solely of observational data. We did not extract identifying information (eg, names, emails) but simple usernames (which are usually pseudonyms). The example pictures (Figure 1 and Figure 2) are licensed under the Creative Commons license and identifying details of the users have been removed.

## Results

### Posting Volume

**Figure 3:**
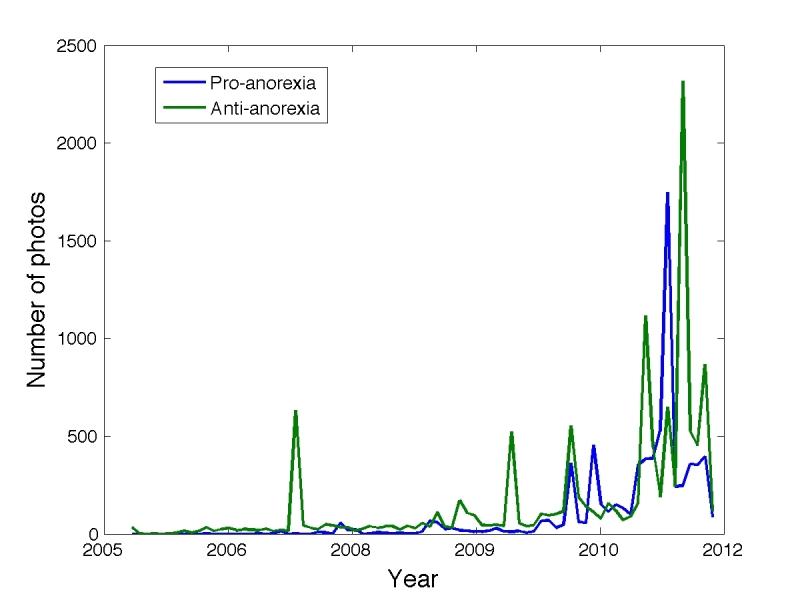
Number of highly relevant photos posted per month (not cumulative, just per month), divided by pro-ana and pro-recovery.

Figure 3 shows the number of highly relevant photographs per month, separated by class. As the figure shows, both types of content are similar in volume and have grown rapidly since 2009. The Spearman correlation between the two time series is 0.82 (*P*< 10^-5^), demonstrating an extremely high correlation. In general, pro-recovery users are more active, posting a median of 196 photos, compared to 105 photographs by pro-anorexia users (statistically significant, ranksum, *P*< 10^-5^).

We examined tags that identify the photo as pertaining to the photographer him or herself. These included the tags “self”, “self-portrait”, and “me”. Pro-anorexia users are responsible for 24% of photographs with these tags, which is a low proportion considering that 40% of the photographs are posted by these users. However, these tags appear in 42% of the highly relevant photos (where 40% of the photographs are posted by pro-anorexia users). Therefore, pro-recovery users generally tend to post more photographs of themselves. When dealing with anorexia-related issues, both pro-anorexia and pro-recovery users are similarly interested in their own images.

### Most Indicative Tags

We computed the probability of using a tag by users from each class (pro-anorexia and pro-recovery) and found the tags for which the ratio of probabilities was highest in each of the classes. The tags with the highest probability for usage by pro-anorexia users, compared to pro-recovery users, were (in descending order of probability ratios): “thinspiration,” “doll,” “thinspo,” “skinny,” “thin,” “cigarette,” “sexy,” “landscape,” “legs,” “abstract,” “long,” “day,” “street,” “body,” “blonde,” “sister,” “nikon,” “up,” “life,” and “model.” For pro-recovery users, these words were: “home,” “sign,” “selfportrait,” “glass,” “cars,” “plants,” “building,” “mother,” “sunshine,” “bird,” “plant,” “autumn,” “garden,” “female,” “fence,” “dog,” “warm,” “architecture,” “stone,” and “birds.”

Therefore, the most important tags for pro-anorexia users refer to body image. Also of note is the use of the tag “cigarette”, which is frequently cited as a way to decrease hunger in the pro-anorexia community. Pro-recovery users have a more varied set of tags, for which we did not discover an underlying theme.

### Inter- and Intra-Community Connectivity

Contacts are more likely in the same class: 72% of contacts by pro-recovery users were to users of the same class, while 59% of the contacts by pro-anorexia users were to users of the same class (*P*< 10^-5^, chi^2^test). Similarly, comments are more likely in-class than between classes, with 83% of the comments by pro-recovery users and 74% of comments by pro-anorexia users being made to users of the same class (*P*< 10^-5^, chi^2^test).

Pro-recovery users are as likely to favorite a photo regardless of the posting users’ stance (56% vs. 44%), but pro-anorexia users are 8.4 times more likely to favorite a photo posted by a pro-anorexia user than by an pro-recovery user (89% vs. 11%, statistically significant at *P*< 10^-5^, chi^2^test).

We modeled the tags used by users to describe their photos using a vector-space model weighted by the Inverse Document Frequency (TF-IDF) [29] and measured the distance between photos using cosine similarity. The average similarity of tags between photographs made by pro-anorexia users was 0.259, between pro-recovery users 0.202, and between the tags of pro-anorexia and pro-recovery users 0.225 (differences significant at *P*< 10^-5^, ranksum test). Therefore, the similarity between pro-recovery and pro-anorexia users is greater than within pro-recovery users. This is partly because pro-recovery users have a broader range of interests (as noted above), but also because pro-recovery users often use tags associated with the pro-anorexia camp: the tag “thinspiration” and its variations are used by 36.8% of pro-anorexia users and by 6.6% of the pro-recovery users. Even more striking is that the tag “pro-anorexia” (and its variations) are used by 1.7% of pro-anorexia users, but 2.4% of pro-recovery users. Overall, the Spearman correlation between tag frequencies in both communities is 0.67 (*P*< 10^-5^).

**Figure 4:**
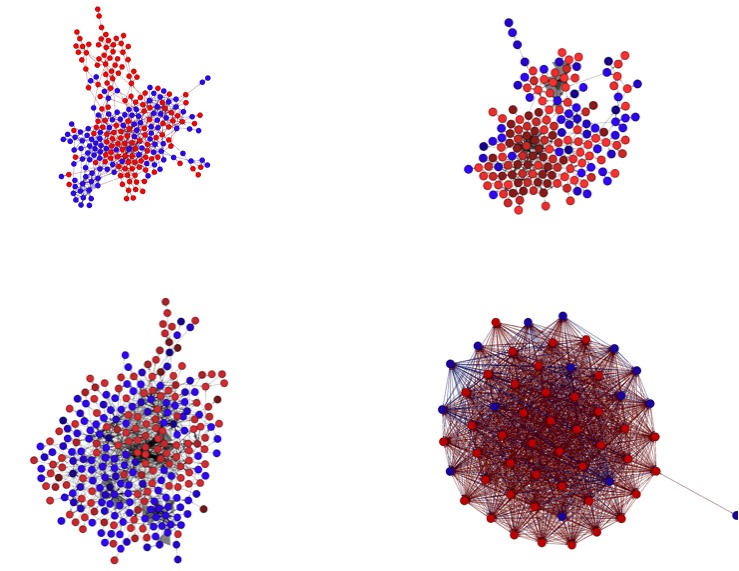
Network graphs according to four connection types (from top left, clockwise): Contacts, Favorites, Tags, Comments.

Figure 4 shows the networks according to the four types of connections between users. Each node is a user. Blue represents a pro-recovery user, and red represents a pro-anorexia user. Only the main connected component is shown in each graph. For tags, we considered two users to be similar in their tags if the cosine distance between their tags was greater than 99%, so as to obtain a sparsity level similar to that of the three other networks. As evident from the graphs, the two classes are intermingled, but are most highly so when observing tags. In order to estimate the separation between classes according to the different networks, we labeled each user according to the difference between the numbers of his or her neighbors of each class. The predictive ability of each network was estimated by the area under the ROC curve [30]. The ROC using the comments or contacts was 0.74, whereas the area using favorites was 0.53 and 0.52 using the tags network. Thus, comments from people of a given class and the class of one’s contacts are the best predictor of one’s class.

### Inter-Community Posting as an Intervention

In order to measure the correlation between attributes of social interaction with the cessation of posting pro-anorexia or pro-recovery photographs (while continuing to post other content on the site), we identified users who stopped posting relevant (either pro-recovery or pro-anorexia) content but continued to post other photographs. We defined posting cessation as stopping posting highly relevant pictures for 3 months or more, while continuing to post other pictures. This definition was used in order to ascertain that the user did not abandon the site (which could happen for several reasons) but continued to use the site for other purposes. In order to measure the effect of comments on cessation, we measured, for each image, whether it was followed by a posting cessation.

The cessation rates and the average days to cessation are shown in Table 1. As the table shows, comments by pro-anorexia users have the same effect on both types of users. However, comments by pro-recovery users decrease cessation in pro-anorexia users and increase it in pro-recovery users. This is also evident in the average days to cessation, which are higher for pro-anorexia users when comments are from the opposite camp, but converse for the pro-recovery camp.

**Table 1:** Rates of stopping posting highly relevant photos by pro-anorexia (PA) and pro-recovery (PR) users.

		Cessation rate		Average days to cessation	
		Commented by…		Commented by…	
		PA	PR	PA	PR
**Posting user**					
	PA	61%	46%	225	329
	PR	61%	71%	366	533

Another way to quantify these effects is through the use of a Cox hazard regression model [31]. For each of the classes, we built a separate model, where the attributes were the log-transformed list given in Tables 2 and 3, normalized to zero mean and unit variance. We computed these features at a resolution of 10 days and attempted to predict the hazard of posting a highly relevant image in the following days.

**Table 2:** Hazard model coefficients; all previous times.

	Class	
	Pro-anorexia	Pro-recovery
Number of highly relevant photos	-0.223^a^	0.013
Number of views	0.212^a^	-0.072^a^
Number of views of highly relevant photos	0.164^a^	0.023
Number of comments from same-class users	-0.057	0.057^a^
Number of comments from other-class users	-0.117^a^	-0.247^a^
Fraction of comments from same-class users	-0.268^a^	-0.027

^a^These numbers are statistically significant at *P*< .05.

**Table 3:** Hazard model coefficients; recent features (calculated using data from the preceding 30 days).

	Class	
	Pro-anorexia	Pro-recovery
Number of highly relevant photos	-0.022	-0.094^a^
Number of views	0.029	0.163^a^
Number of views of highly relevant photos	-0.007	0.199^a^
Number of comments from same-class users	-0.068^a^	0.024
Number of comments from other-class users	0.057^a^	-0.002
Fraction of comments from same-class users	0.061^a^	0.172^a^

^a^These numbers are statistically significant at *P*< .05.

The results of this model show that pro-anorexia users are encouraged to post additional photographs when many people view the photos they have posted and, in the short term, by comments of the pro-recovery group. Pro-recovery users are encouraged by viewings and by comments of their own group but discouraged by comments from pro-anorexia users.

## Discussion

The manifestation on the web of anorexia nervosa content can be divided into pro-recovery content, supporting people trying to recover from the disease, and pro-anorexia content, which supports and even encourages people to continue their current behavior. In this paper, we have investigated these two communities and the interactions between them. These two communities interact by means of users changing from one community to the other and more generally by commenting on, following, and browsing the content from the antagonist community.

We found that comments and contacts are more likely in-community than between communities. Favorites by pro-recovery users are equally likely to be members of the two communities, but pro-anorexia users are 8.4 times more likely to favorite pro-anorexia photos. Taken together, these show two active communities of users, who mostly interact in their own community. The main divergence from this behavior is in marking favorite photographs. This is most likely because the receiving user can delete comments and contacts but not favorite markings. Therefore, it is likely that the figures for comments and contacts are following filtering by the receiving user.

Our results show that the two communities coexist separately according to the (moderated) comments and contacts, but interestingly, they are intermingled according to the tags and (unmoderated) favorite links. A possible explanation for this is that the pro-recovery group is trying to expose itself to pro-anorexia users through the use of similar tags, thus causing images posted by them to surface in search of the pro-anorexia users. The reason for this is that search for images is conducted by comparing the user’s query with the tags as well as the textual description of the image. Furthermore, by marking as favorites pro-anorexia images, they may be causing the users who posted them to be exposed (indirectly) to pro-recovery content.

However, by modeling the likelihood of discontinuing posting photos, we found that an intervention of comment posting by pro-recovery users is counter-productive, causing pro-anorexia users to continue posting for longer and, if they cease posting, to do so later. Previous studies have found that pro-anorexia users perceive themselves as isolated in the physical world [32]. It may be that pro-recovery comments reinforce this feeling, entrenching users in their behavior.

Thus, pro-recovery users undertake two kinds of interventions. First, they expose pro-anorexia users who search for pro-anorexia content to pro-recovery content. Second, they post comments to pro-anorexia content. The latter, at least, is detrimental, as measured by the cessation of posting.

There are several unique aspects to our study, which is based on a social photo-sharing site, that need to be taken into account since they do not necessarily generalize to all pro-anorexia websites. Flickr is a general-purpose photo-sharing platform where users can easily create their own groups, and there are nearly no limitations on the interaction between different communities. In this context, pro-anorexia and pro-recovery communities have to co-exist on the platform and therefore interactions are likely to happen. Previous studies of pro-anorexia communities have been performed in online forums used exclusively by the pro-anorexia community [3-5,11-13,17,18]. Our study findings cannot be generalized to all types of pro-anorexia sites, but they are most likely to be similar within general social networks where pro-recovery and pro-anorexia users are more likely to interact.

In addition to the risks of pro-anorexia webs reviewed by Rouleau et al [14], we identified other risks inherent from the interaction of pro-anorexia and pro-recovery users. Individual pro-anorexia users have the possibility of influencing members of pro-recovery communities. As explained below, in this highly dynamic social network environment to fully understand the communities’ interactions is of vital importance so as to reduce the influence of pro-anorexia users and content.

### Clinical Relevance

The understanding of these communities is of key interest to public health officials aiming to prevent anorexia and minimize the impact of pro-anorexia content online. Our results show that the pro-anorexia and pro-recovery communities in Flickr have grown in their volume in a similar manner over time. Consequently, it is important to study the two communities and the interactions between them. That information can be extracted automatically as we did in this study in order to enact surveillance of the prevalence of the community online. As shown in this study, there are cases where pro-anorexia users actively try to persuade members of pro-recovery communities. Public health interventions on content-based social platforms, such as Flickr and YouTube, must be aware of the possibility that social network dynamics may undermine the effects of their intervention. Burton highlighted the importance of public health community mining for public health professionals [33]. In fact, to understand the dynamics of health communities, it is of vital importance to design online social methods for health promotion [34].

Automatic analysis of the pro-anorexia communities can be used to improve online interventions, such as warning messages that have been already piloted with promising results [11]. The suggested intervention by Lewis and Arbuthnott [17] to prevent access to pro-anorexia websites from the search results can benefit from our findings about the different tagging patterns used by the pro-anorexia community. An online intervention aimed at people affected by anorexia nervosa can be displayed when users are searching for pro-anorexia related terms. In addition, the network dynamics described in this paper can guide public health officials disseminating content in social networks at how to gain more visibility and reputation within the different online communities.

Our study provides deeper understanding of the online behavior of people affected by eating disorders. Clinicians should consider assessing the online activity of their patients to identify contributing factors, such as engagement in pro-anorexia communities, and provide guidelines about a safe use of the Internet since a simplistic approach based on banning Internet will limit access to trustworthy health information and to support from pro-recovery communities.

Similar approaches have been suggested in the area of online content related to self-injury [35,36]. Self-injury online content is currently starting to raise concerns due to its pernicious nature [8,35-38].

### Limitations

Our study has several limitations: First, our study is limited to communities within Flickr (as discussed above). Second, while users identified by manual labeling are highly likely to be correctly assigned to their respective community (high precision), our collection method does not ensure high recall, ie, we are likely missing users who were not identified by our collection method. Third, as our data collection relied on public APIs, we have very little interaction data such as specific viewing behavior of users. Thus, for example, users who only browse content but never post any photo, comment, or favorite link will be missing from our data, as will the effect of such viewing on individual behavior. Finally, our data does not contain any clinical indication of users’ actual state. This is especially evident in using posting cessation as a measure of engagement, where actual information on recovery or otherwise would have been of immense value. However, privacy concerns make it unlikely that such ground-truth labels can be obtained.

Future work will focus on identifying successful intervention strategies, tailored to the traits of specific users. We envision a system that will learn the particular characteristics of each user (for example, distinguishing between first-time viewers and active pro-anorexia content contributors) in order to apply one of a set of possible interventions, including warning labels, content blocking, reports to guardians (as in parental control software), or semi-automated comments to posting of pro-anorexia content. In addition, we will explore if our findings are applicable to other online communities based on a different type of content (eg, videos). We expect our results to be similar in other communities based on sharing multimedia content such as images and videos.

### Conclusions

Our investigation of photo sharing behavior by the pro-anorexia community and the pro-recovery community has uncovered significant differences between the two. Better understanding of the pro-anorexia community can guide public health officials designing online interventions for people at risk of eating disorders or to mitigate the effects of such communities on individuals. In addition, our study is a first step towards the design of advanced filtering tools that will prevent pro-anorexia content from reaching vulnerable individuals.

## References

[1] Hoek HW, van Hoeken D (2003). Review of the prevalence and incidence of eating disorders. Int J Eat Disord.

[2] Arcelus J, Mitchell AJ, Wales J, Nielsen S (2011). Mortality rates in patients with anorexia nervosa and other eating disorders. A meta-analysis of 36 studies. Arch Gen Psychiatry.

[3] Norris ML, Boydell KM, Pinhas L, Katzman DK (2006). Ana and the Internet: a review of pro-anorexia websites. Int J Eat Disord.

[4] Borzekowski DL, Schenk S, Wilson JL, Peebles R (2010). e-Ana and e-Mia: A content analysis of pro-eating disorder Web sites. Am J Public Health.

[5] Mulveen R, Hepworth J (2006). An interpretative phenomenological analysis of participation in a pro-anorexia internet site and its relationship with disordered eating. J Health Psychol.

[6] Kummervold PE, Chronaki CE, Lausen B, Prokosch HU, Rasmussen J, Santana S, Staniszewski A, Wangberg SC (2008). eHealth trends in Europe 2005-2007: a population-based survey. J Med Internet Res.

[7] Fox S, Jones S (2009). The social life of health information.

[8] Ache KA, Wallace LS (2008). Human papillomavirus vaccination coverage on YouTube. Am J Prev Med.

[9] Mitchell KJ, Ybarra ML (2007). Online behavior of youth who engage in self-harm provides clues for preventive intervention. Prev Med.

[10] Linkletter M, Gordon K, Dooley J (2010). The choking game and YouTube: a dangerous combination. Clin Pediatr (Phila).

[11] Wilson JL, Peebles R, Hardy KK, Litt IF (2006). Surfing for thinness: a pilot study of pro-eating disorder Web site usage in adolescents with eating disorders. Pediatrics.

[12] Custers K, Van den Bulck J (2009). Viewership of pro-anorexia websites in seventh, ninth and eleventh graders. Eur Eat Disord Rev.

[13] Harper K, Sperry S, Thompson JK (2008). Viewership of pro-eating disorder websites: association with body image and eating disturbances. Int J Eat Disord.

[14] Rouleau CR, von Ranson KM (2011). Potential risks of pro-eating disorder websites. Clin Psychol Rev.

[15] BBC News, Israel passes law banning use of underweight models.

[16] Thinspiration: Doctors Concerned With Social Media Sites Promoting Eating Disorders.

[17] Lewis SP, Arbuthnott AE (2012). Searching for thinspiration: the nature of internet searches for pro-eating disorder websites. Cyberpsychol Behav Soc Netw.

[18] Martijn C, Smeets E, Jansen A, Hoeymans N, Schoemaker C (2009). Don't get the message: the effect of a warning text before visiting a proanorexia website. Int J Eat Disord.

[19] (2012). Flickr Statistics.

[20] Alexa Internet Inc, Flickr Site Info, 2012, www com/siteinfo/flickr.

[21] (2012). Thinspiration.

[22] (2009). Anorexia Nervosa.

[23] Flickr API Services.

[24] Eating Disorders Art Group.

[25] Anorexia Nervosa Group.

[26] Anorexia Help.

[27] ED Healing.

[28] Cohen J (1960). A Coefficient of Agreement for Nominal Scales. Educational and Psychological Measurement Internet.

[29] van Rijsbergen CJ (1979). Information Retrieval. 2nd Edition. Butterworths.

[30] Duda RO, Hart PE, Stork DG (2001). Pattern Classification. 2nd ed.

[31] Allison P (1995). Survival Analysis Using the SAS System: A Practical Guide.

[32] Gavin J, Rodham K, Poyer H (2008). The presentation of "pro-anorexia" in online group interactions. Qual Health Res.

[33] Burton S, Morris R, Dimond M, Hansen J, Giraud-Carrier C, West J (2012). Public health community mining in YouTube Internet. Proceedings of the 2nd ACM SIGHIT symposium on International health informatics - IHI '12.

[34] Gosselin P, Poitras P (2008). Use of an internet "viral" marketing software platform in health promotion. J Med Internet Res.

[35] Lewis SP, Heath NL, Michal NJ, Duggan JM (2012). Non-suicidal self-injury, youth, and the Internet: What mental health professionals need to know. Child Adolesc Psychiatry Ment Health.

[36] Whitlock J, Lader W, Conterio K (2007). The internet and self-injury: what psychotherapists should know. J Clin Psychol.

[37] Lewis SP, Heath NL, St Denis JM, Noble R (2011). The scope of nonsuicidal self-injury on YouTube. Pediatrics.

[38] Lewis SP, Baker TG (2011). The possible risks of self-injury web sites: a content analysis. Arch Suicide Res.

